# Attitudes and Practices of Dental Hygienists Regarding Diabetes Screening and Medical–Dental Collaboration: A Nationwide Cross-Sectional Study in Japan

**DOI:** 10.3390/healthcare13172174

**Published:** 2025-08-30

**Authors:** Rie Kudoh, Taiga Shibayama

**Affiliations:** Institute of Medicine, University of Tsukuba, 1-1-1 Tennoudai, Tsukuba-shi 305-8575, Ibaraki, Japan; taiga@md.tsukuba.ac.jp

**Keywords:** cross-sectional study, dental hygienist, diabetes, medical–dental collaboration, screening

## Abstract

**Background/Objectives**: Despite the bidirectional relationship between diabetes and periodontal disease, dental hygienists’ role in diabetes screening remains underexplored in Japan. Previous studies have not examined the relationship between attitudes and actual screening practices. This study aimed to assess dental hygienists’ attitudes regarding diabetes screening and medical–dental collaboration, examine current practices, and investigate the association between attitudes and practices. **Methods**: A nationwide cross-sectional survey was conducted among dental hygienists from 1340 dental clinics using stratified random sampling. Participants completed an anonymous questionnaire assessing attitudes (4-point Likert scale) and screening practices (4-point frequency scale). Exploratory factor analysis and multiple regression analysis examined attitude–practice associations. **Results:** Of 1340 surveys distributed, 95 valid responses were obtained (response rate: 7.2%). Participants showed low implementation rates for lifestyle and physical sign assessment in diabetes (below 35%, except thirst: 68.4%) but moderate-to-high rates for medical history evaluation (44.2–75.8%). Multiple regression analysis revealed that lack of confidence and knowledge in diabetes management was negatively associated with both lifestyle assessment (β = −0.38; 95% CI −0.72 to −0.23) and medical history evaluation (β = −0.55; 95% CI −0.63 to −0.32). Desire to participate in diabetes education was positively associated with medical history evaluation (β = 0.21; 95% CI 0.24 to 4.38). **Conclusions**: Despite low response rate limiting generalizability, this first nationwide Japanese survey indicates that confidence and knowledge deficits significantly hinder diabetes screening practices. Targeted educational interventions could enhance screening implementation and strengthen medical–dental collaboration, improving early diabetes detection.

## 1. Introduction

Diabetes is a chronic metabolic disease characterized by inflammation and hyperglycemia. It carries high mortality and morbidity risks due to long-term complications, including cardiovascular disease, diabetic retinopathy, and kidney failure [1]. Nearly one million people globally are projected to develop diabetes each year, with approximately 80% of those already affected dying from its complications [2]. In Japan, approximately 20 million individuals are affected by diabetes and pre-diabetes [3]. Therefore, prevention of these complications is essential.

Currently, approximately 60% of the Japanese population is affected by periodontal disease [4]. Several studies have demonstrated an association between diabetes and periodontal disease, suggesting a bidirectional relationship between both pathologies [5,6,7]. Diabetes mellitus represents a significant risk factor for developing and worsening periodontal disease. It has been observed that people with diabetes present a significantly increased prevalence of chronic or severe periodontal diseases compared to non-diabetic people, with the risk of periodontal disease progression increasing approximately threefold [8,9]. Periodontal disease can lead to substantial tooth loss, impaired mastication, nutritional deficiencies, and reduced quality of life [2,10]. Furthermore, it can contribute to the development of insulin resistance, worsen glycemic control, and may affect the onset and progression of diabetes complications, such as retinopathy, neuropathy, nephropathy, and cardiovascular disease [5,11]. The multifaceted impact of periodontal disease underscores its significance as a public health concern [12]. Given these two-way relationships, care and patient education in medicine and dentistry are needed to improve oral health in people with diabetes [13].

The utility of diabetes screening in dental settings and referral to primary care as a means to improve the diagnosis of prediabetes and diabetes has been reported [14]. It has been estimated that approximately 30% of people aged 30 years or older seen in general dental practices have dysglycemia [14]. Thus, the assessment of peoples’ risk for prediabetes and type 2 diabetes mellitus during dental hygiene assessments is recommended as a standards practice for dental hygienists (DHs) in the United States [15]. Screening using the American Diabetes Association Diabetes Risk Test has been positively correlated with HbA1c levels in people receiving periodontal maintenance care [16,17]. Screening conducted by DHs has been suggested to be effective and convenient for identifying undiagnosed prediabetes and for providing opportunities for interprofessional patient care [16]. Also, DHs were aware of the association between periodontal disease and diabetes and felt that it was important to practice chairside screening for diabetes were reported [18]. These efforts by DHs may support early detection and timely referral to medical professionals, thereby potentially strengthening medical–dental collaboration and contributing to more comprehensive diabetes management [18]. This practice can ultimately support early diagnosis and potentially lessen the economic burden of diabetes. However, despite the recognized importance of such collaborative approaches, implementation remains challenging. A previous review reported that although approximately 70% of DHs recognized the need for medical–dental collaboration in the oral management of patients with diabetes, only about 30% were actively collaborating with medical professionals [18]. This gap between recognition and practice may reflect historical barriers to interprofessional collaboration. For many years, oral health has been regarded as the responsibility of dentists and treated as separate from general health, despite its close relationship to it, reflecting a longstanding divide between the medical and dental professions [19]. Furthermore, unlike countries such as the United States where DHs have expanded scope of practice including risk assessment and patient counseling, Japanese regulations restrict DHs to preventive oral healthcare under direct dentist supervision [18]. These regulatory constraints may further limit the potential for DHs to engage in diabetes screening and interprofessional collaboration.

However, the association between attitudes and practices related to diabetes screening and medical–dental collaboration in dental settings has not been thoroughly examined among DHs in Japan. Therefore, this study aimed to assess DHs’ attitudes regarding diabetes screening and medical–dental collaboration, examine current practices, and investigate the association between attitudes and practices.

## 2. Materials and Methods

### 2.1. Design

This descriptive study employed a cross-sectional survey design using an anonymous web-based questionnaire conducted at the national level in Japan. A stratified random sampling method was used to recruit DHs from dental clinics across Japan. The study was approved by the ethics committee of the authors’ affiliated university (approval number: 2054; approval date: 25 October 2024). A written explanation of the survey was included in the request letter. Consent was provided by respondents upon return of the completed survey.

### 2.2. Participants

The participants in this study were DHs working at 1340 dental clinics registered with the Japan Dental Association. Inclusion criteria were being employed at a dental clinic listed on the publicly available dentist search website of the Japan Dental Association as of 15 July 2024. Exclusion criteria included DHs who did not provide consent to participate in the survey or were not working at the clinic at the time of completing the questionnaire.

### 2.3. Sampling and Recruitment

The target population comprised dental clinics listed on the dentist search website publicly available on the Japan Dental Association homepage [20], as of 15 July 2024. From a total of 52,604 registered facilities, 1340 clinics were selected as the study sample using stratified random sampling. Stratified random sampling was conducted using all 47 prefectures as strata. The number of facilities selected from each prefecture was proportionally allocated based on the prefecture’s share of the national total of dental clinics (range: 1–85 facilities per prefecture, reflecting the distribution from rural to metropolitan areas). This weighting approach ensured that prefectures with larger numbers of dental clinics contributed proportionally more samples, while those with fewer clinics contributed fewer samples, thereby maintaining the relative distribution of dental clinics across Japan as observed in the source population. The required sample size was calculated, assuming an effect size of 0.15, statistical power of 0.80, and a significance level of 0.05. The calculation indicated that a minimum of 268 facilities would be required for adequate statistical power. Considering an anticipated response rate of 20%, questionnaires were distributed to 1340 dental clinics, with a target response of 268 facilities. The participants were recruited between October 2024 and January 2025.

### 2.4. Procedures

An anonymous web-based questionnaire was administered to DHs employed at the selected dental clinics. Survey materials, including a cover letter, the questionnaire URL, and a QR code, were mailed to each of the 1340 target facilities (one set per facility). The cover letter included a request for clinic administrators to distribute the survey participation invitation to DHs employed at their respective facilities. DHs who worked in dental clinics were included in this study. Participants who were unwilling to participate or did not work at the clinic at the time of answering the questionnaire were excluded from the study.

### 2.5. Measures

Participants’ background information, attitudes, and practices of the participants toward diabetes screening in a dental setting were assessed using a self-reported questionnaire developed for the study. First, the content of the questionnaire was developed based on guidelines, reports, and books related to diabetes and periodontal disease, along with a comprehensive review of previous research findings. Second, items related to DHs’ attitudes and practices regarding diabetes screening and medical–dental collaboration were identified and drafted as questionnaire items. Third, the questionnaire was reviewed and revised through discussions among researchers with expertise in diabetes nursing and DHs. Finally, a pilot test was conducted with 75 eligible DHs who were active members of the Society for Dental Hygiene in the Kanto region, leading to refinement in the questionnaire based on the results. The questionnaire comprised the following:

Background characteristics included age, gender, years of clinical experience as registered DHs, time available for providing education to people with diabetes, working patterns in dental clinics, experience in education related to oral management for people with diabetes, and willingness to participate in future education on oral management of diabetes. Attitudes toward diabetes screening and medical–dental collaboration in a dental setting were assessed using an ordinal scale ranging from 1 (“Strongly disagree”) to 4 (“Strongly agree”). Practices were measured using 15 items related to diabetes screening, assessed on an ordinal scale ranging from 1 (never) to 4 (always). In addition, five items related to symptoms potentially associated with diabetes—“thirst,” “polydipsia,” “polyuria,” “weight loss,” and “fatigue”—were evaluated using the same 4-point ordinal scale, and the scores for each symptom item were used in the analysis. Furthermore, practices related to oral management for people with diabetes were assessed using four items, each evaluated on a binary scale (yes/no).

### 2.6. Statistical Analysis

We described the distribution of quantitative data in terms of mean, standard deviation, percentage, and range. We performed exploratory factor analysis (EFA) on items related to DHs’ attitudes and practices toward diabetes screening and medical–dental collaboration. We performed EFA on the variance covariance matrix with missing values complemented by the Expectation–Maximization (EM) algorithm. For EFA, the number of factors to extract was determined using parallel analysis [21,22]. This method compares the eigenvalues from the actual data with eigenvalues generated from random data sets of the same sample size and number of variables. Factors with eigenvalues exceeding the 95th percentile of random eigenvalues were retained. We extracted the factors using promax rotation of the unweighted least-squares factor solution and calculated the internal consistency of each factor using Cronbach’s alpha coefficients. We also performed multiple regression analyses to examine the association of explanatory variables with DHs’ diabetes screening practices. The explanatory variables included DHs’ attitudes toward diabetes screening and medical–dental collaboration, gender, clinical experience, employment status at dental clinics, time for diabetes patient education at dental clinics, and desire to participate in education regarding oral management of diabetes.

Missing data were imputed using the multiple imputation method for multiple regression analysis. We analyzed the data using SAS 9.4.

## 3. Results

Of the 1340 survey packages mailed to dental clinics, 11 were returned due to unknown addresses and one facility reported closure via telephone contact. A total of 97 participants were obtained from DHs working at the participating dental clinics. Data from one participant who was unwilling to participate in the study and one participant who did not provide valid responses were excluded from the analysis. The overall response rate was 7.2% (97/1340). A total of 95 valid responses were included in the final analysis.

The background characteristics of the participants are presented in Table 1. On average, participants had 20 years of experience working as DHs. Approximately 80% were employed full-time at dental clinics. Parallel analysis was conducted using SAS 9.4 to determine the optimal number of factors. For attitude items, the first two observed eigenvalues (5.33, 2.90) exceeded the corresponding 95th percentile random eigenvalues (2.24, 1.96), indicating a two-factor solution. The results of the EFA of the attitude items are listed in Table 2. Three items with low factor loadings were excluded. Descriptive statistics of the final attitude items are presented in Table 3. Participants’ attitude was highest regarding “It is necessary to assess diabetic status and periodontal treatment risks during oral management.” The lowest attitude scores were for “I do not feel the need for medical–dental collaboration regarding diabetes treatment in daily practice,” followed by “I am confident in diabetes screening” as the second lowest.

Regarding practice, parallel analysis indicated that two factors should be retained for the 20 items assessed as diabetes screening practices (observed eigenvalues: 8.54, 2.35 vs. random eigenvalues: 2.20, 1.93). The EFA of DHs’ practice towards diabetes screening is shown in Table 4. Four items with low factor loadings were excluded. The final solutions are presented in Table 5. Practice of lifestyle background and physical sign assessment in diabetes showed implementation rates below 35%, except for thirst (68.4%).

Participants’ practices for medical history and treatment status evaluation in diabetes showed implementation rates of 44.2–75.8%. Moreover, participants’ practice was highest regarding “Ever been told your blood glucose was high” and “Blood pressure,” and lowest regarding “Body mass index.” The reported rates of practice for the four items related to oral management for people with diabetes were as follows: 68.4% for recommending more frequent regular dental checkups, 36.8% for targeted oral hygiene instruction, 21.0% for dental treatment with consideration of blood glucose levels, and 13.6% for collaboration with nutritional counseling.

**Figure 1:**
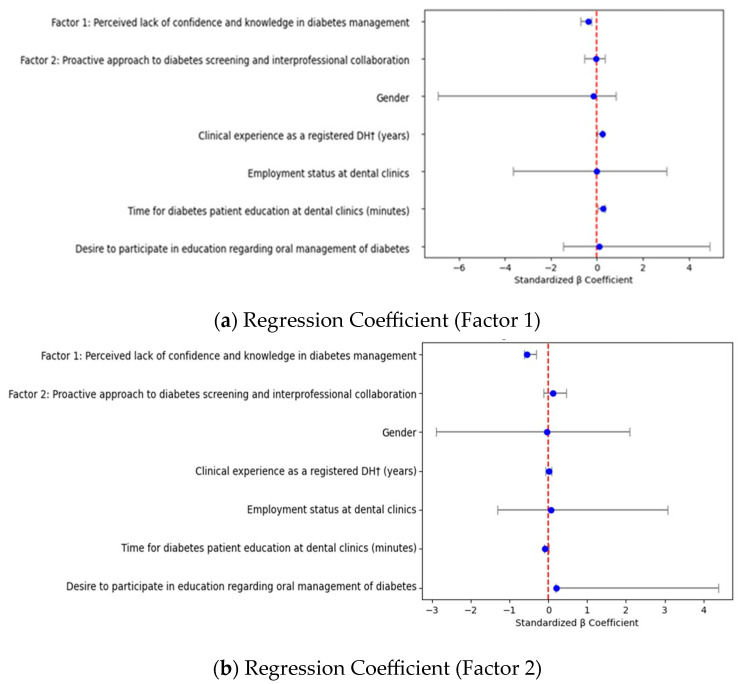
Standardized regression coefficients for Factor 1 and Factor 2 models. Forest plots display standardized partial regression coefficients (β) and 95% confidence intervals for each predictor included in the models. Practices were assessed through two factors: Upper panel (Factor 1) Lifestyle background and physical sign assessment in diabetes and lower panel (Factor 2) Medical history and treatment status evaluation in diabetes. Vertical dashed lines indicate the null value (β = 0).

The results of the multiple regression models of the DHs’ practices toward diabetes screening are shown in Table 6. Figure 1 presents the standardized regression coefficients for Factor 1 and Factor 2 models. This figure allows us to enhance the interpretability of the regression analysis results. We visualized the standardized regression coefficients and their 95% confidence intervals for each predictor variable. Participants who strongly perceived lack of confidence and knowledge in diabetes management were less likely to check lifestyle background and physical sign assessment in diabetes (Factor 1: standardized partial regression coefficient [β] = −0.38; 95% confidence interval [CI] −0.72–−0.23). These participants were also less likely to check medical history and treatment status evaluation in diabetes (Factor 2: [β] = −0.55; 95% confidence interval [CI] −0.63–−0.32), while desire to participate in education regarding oral management of diabetes was positively associated with medical history and treatment status evaluation in diabetes (Factor 2: β = 0.21; 95% CI 0.24 to 4.38). Attitudes toward a proactive approach to diabetes screening and interprofessional collaboration were not statistically significant in the model.

## 4. Discussion

DHs are preventive specialists and expect to play a key role in practicing diabetes screenings and medical–dental collaboration in dental settings [15,18]. We report on the first nationwide survey among DHs to examine their attitudes and practices regarding diabetes screening and medical–dental collaboration in Japan.

Participants’ attitudes scores toward diabetes screening and medical–dental collaboration tended to be relatively high. In particular, the highest scores were observed for “It is necessary to assess diabetic status and periodontal treatment risks during oral management.” In contrast, regarding the actual practices of diabetes screening among these participants, implementation scores for assessing lifestyle background and physical signs in diabetes tended to be lower, except for thirst assessment, while practices related to medical history and treatment status evaluation showed moderate to relatively high implementation levels. The consistently low implementation rates for lifestyle and physical sign assessment (below 35%) represent a significant missed opportunity for early diabetes detection. Given that approximately 30% of dental patients aged 30 years or older have dysglycemia, these low screening rates suggest that a substantial number of undiagnosed cases may be overlooked in dental settings annually. Previous studies have reported that topics with a less immediate or direct relationship to oral health—such as glycemic control, nutrition and dietary counseling, and physical activity/exercise—tended not to be addressed [23,24]. While diabetes is characterized by asymptomatic features in its early stages, symptoms such as thirst, polydipsia, polyuria, weight loss, and fatigue develop as the disease progresses [14]. Therefore, assessing physical signs together with lifestyle background that may be associated with diabetes is essential for screening people who are suspected of having diabetes and those who are likely candidates for diabetes. One possible explanation for the higher score for thirst assessment is that this symptom is immediate and direct connection to oral health.

In addition, present findings suggested that participants had lower levels of confidence in diabetes screening and perceived need for medical–dental collaboration in daily practice. Although DHs have been encouraged to screen periodontal patients for diabetes, they had no confidence performing diabetes screening [15]. Additionally, it has been reported that over 70% of DHs acknowledged the need for medical–dental collaboration in the oral management of people with diabetes and expressed a willingness to be involved in interprofessional collaborative care on this issue [13,25]. However, the actual practice of such collaboration remained limited, with only 30% of DHs engaging in communication regarding a people’s glycemic control was reported [26]. Therefore, the findings suggest the need for strategies to enhance DHs’ levels of confidence in diabetes screening, promote the practices of assessing lifestyle background and physical signs in diabetes, and strengthen the actual practices of medical–dental collaboration in dental settings.

Regarding the practices related to oral management for people with diabetes, nearly 70% of participants reported practicing the recommendation of more frequent regular dental checkups. While the implementation rates for targeted oral hygiene instruction, dental treatment with consideration of blood glucose levels, and collaboration with nutritional counseling tend to be lower. To promote the practices of targeted oral hygiene instruction and dental treatment with consideration of blood glucose levels, collaboration with medical professionals is essential. It has been pointed out that training for both medical and dental professionals could facilitate the oral management of people with diabetes through medical–dental collaboration [27,28]. For example, this type of training could involve equipping medical professionals to screen patients with diabetes for oral problems, and dental professionals to identify the risk factors for systemic diseases, including diabetes [25]. Accordingly, it is necessary to provide such interprofessional education to promote effective medical–dental collaboration in the management of diabetes. Surprisingly, 13.6% of dental clinics reported practicing collaboration with nutritional counseling. The present study targeted standalone dental clinics and did not include hospital-affiliated dental departments. Nevertheless, it is noteworthy that some dental clinics have made progress in establishing collaboration with nutritional counseling, although limited in number. There is a need to increase the number of such facilities in the future.

The present study found that participants who perceived lack of confidence and knowledge in diabetes management were negatively associated with both lifestyle background and physical sign assessment in diabetes and medical history and treatment status evaluation in diabetes, while desire to participate in education regarding oral management of diabetes was positively associated with medical history and treatment status evaluation in diabetes. The strong negative associations between lack of confidence/knowledge and screening practices (β = −0.38 for lifestyle assessment, β = −0.55 for medical history evaluation) indicate that educational interventions targeting these specific barriers could substantially improve implementation rates. The larger effect size for medical history evaluation suggests that knowledge-based interventions may be particularly effective in this domain. Attitudes toward a proactive approach to diabetes screening and interprofessional collaboration were not statistically significant in the model. Based on these findings, targeted educational interventions should focus on the following: (1) hands-on training for diabetes risk assessment techniques, (2) case-based learning to build confidence in patient screening, (3) interprofessional collaboration workshops with medical professionals, and (4) development of standardized screening protocols for dental settings. The positive association between desire for education and practice implementation (β = 0.21) suggests that motivated learners would benefit significantly from such programs.

The Japanese healthcare context presents unique structural challenges for diabetes screening practice in dental settings. While the healthcare system has established reimbursement pathways for medical practitioners to refer diabetes patients to dental care, no corresponding financial incentives exist for dental practitioners to conduct diabetes screening or refer patients to medical care. This creates an asymmetric referral system that may limit organizational motivation for dental clinics to invest in diabetes screening capabilities. Furthermore, unlike countries such as the United States where DHs have expanded scope of practice including risk assessment and patient counseling, Japanese regulations restrict DHs to preventive dental care under direct dentist supervision [29]. This regulatory framework may explain why positive attitudes toward diabetes screening did not translate to actual practice implementation, as DHs may lack the professional autonomy to initiate systematic screening protocols or patient referrals independently. In its 2024 revision of medical service fees, the Ministry of Health, Labour and Welfare in Japan recommended that patients with diabetes undergo dental consultations [30]. However, diabetes screening in dental settings does not yet have corresponding medical service fees. Previous review pointed out that organizational support depends on the payment of medical fees [18]. Evidence for diabetes screening in dental settings remains limited and under development. To facilitate the integration of such practices into the medical fee schedules, more data on intervention effectiveness is required.

In the United States, chairside HbA1c testing has been introduced as part of diabetes screening in dental settings. More than 80% of dentists reported willingness to provide HbA1c screening in dental clinics [31], whereas only 24% of DHs expressed willingness to do so [15]. However, in Japan, many dentists are not supportive of practicing chairside HbA1c testing because it is not covered by the dental reimbursement system, and no data are available from DHs regarding their perspectives on this matter. Furthermore, the legal scope of DHs’ duties in Japan is limited to three areas: preventive treatment, assistance with dental procedures, and provision of health guidance, all under the supervision of dentists [29]. This restricted professional autonomy likely influences the role of DHs in diabetes management within dental settings and the applicability and feasibility of diabetes screening such as chairside HbA1c testing in Japan.

In our previous literature review, we compared dental hygienists’ attitudes and practices toward oral management for people with diabetes in Japan with findings in the United States [18]. Only nine articles met the inclusion criteria, suggesting that research in this field remains limited in both countries. Comparing DHs’ attitudes and practices toward diabetes screening and medical–dental collaboration of Japanese DHs in this study and the United States in previous review revealed several key differences and similarities. Japanese DHs in this study reported relatively high attitude scores toward diabetes screening and medical–dental collaboration. Similarly, over 70% of DHs in the United States demonstrated understanding of the diabetes–periodontal disease link, although some reported knowledge gaps in screening practices. Regarding clinical practices, Japanese DHs showed lower implementation scores for assessing lifestyle and physical signs, except for thirst assessment, whereas evaluations of medical history and treatment status showed moderate to high implementation levels. DHs in the United States emphasized patient education and comprehensive care, though implementation varied among practitioners. Both countries recognized the importance of medical–dental collaboration. These differences likely reflect distinct healthcare systems and professional roles. In the United States, dental hygiene encompasses recognition, prevention, and treatment of oral diseases as part of total health [32]. DHs are responsible for disease prevention and health promotion while independently conducting assessments, making clinical decisions, and initiating treatment without dentist authorization [33].

Collectively, these findings highlight how differences in professional scope and healthcare system structures shape DHs’ roles in diabetes management. They also provide contextual foundation for understanding the current situation of DHs in Japan and evaluating the potential for practicing chairside diabetes screening in Japanese dental practice.

Our results suggest that DHs may need proper education to improve knowledge and confidence in diabetes screening to incorporate these practices into the process of care. Previous studies have identified barriers to DHs’ practice in oral management of diabetes, including a lack of education of DHs, a lack of instructional materials for patient education, and patient acceptance [15,26,34]. Consequently, it is suggested that educational opportunities be enhanced and instructional materials, including e-learning resources, be developed to promote the practice of oral management in diabetes care. The Ministry of Health, Labour and Welfare (2024) also announced issues related to collaboration between medical, pharmaceutical, and dental sectors in Japan [30]. These findings have direct implications for healthcare policy and professional education. We recommend: (1) inclusion of diabetes screening competencies in dental hygienist curricula, (2) development of evidence-based screening protocols for dental settings, (3) establishment of medical service fees for diabetes screening activities in dental clinics, and (4) creation of formal referral pathways between dental and medical professionals. Professional dental organizations should develop continuing education programs focused on diabetes risk assessment, while educational institutions need to integrate medical–dental collaboration training into their curricula. Therefore, further strategies for medical–dental collaboration in Japan are warranted, in addition to the education of DHs and the development of organizational support systems. If diabetes screening were practiced by more DHs across a range of dental clinics, with appropriate referral to medical professionals, it may improve the diagnosis of prediabetes and diabetes. This could be useful for preventing diabetes complications, ultimately contributing to the maintenance of people’s quality of life.

The present study has several limitations. First, while the 7.2% response rate limits generalizability, this low participation rate itself provides important insights into the current state of diabetes-related care awareness in Japanese dental settings. The limited response may reflect insufficient recognition of the importance of medical–dental collaboration in diabetes management, further emphasizing the urgent need for educational initiatives and awareness campaigns. Additionally, the participants who responded may represent individuals with relatively high interest in the oral management of people with diabetes, introducing potential selection bias. Since the valid responses were fewer than those calculated by the sample size, the data must be interpreted with caution and cannot be extrapolated. Furthermore, the very low response rate raises the possibility of non-response bias, as non-respondents may differ systematically from respondents in their awareness, attitudes, or practices related to oral management in diabetes care. These limitations underscore the need to interpret the findings cautiously and avoid overinterpreting the results. Future studies should consider strategies to improve response rates, such as follow-up reminders, incentives for participation, or simplified survey procedures, to obtain more representative samples. The achieved sample size (*n* = 95) was far below the originally planned *n* = 268, which also reduces confidence in the results, including the factor analyses, and should be considered a major limitation of the study. This limitation further underscores the need to interpret the findings cautiously and highlights the importance of future studies with higher response rates and more representative samples. In addition, most participants in this study were female, reflecting the gender distribution of DHs in Japan. According to the Japan Dental Hygienists’ Association’s 2025 “Report on the Working Conditions of Dental Hygienists,” 99.5% of DHs are female and 0.5% are male [35]. While this may limit the generalizability of the findings to male DHs, the impact on the overall results is likely minimal due to the small proportion of males in the profession. Future studies could examine potential gender differences in diabetes screening practices and attitudes. Moreover, this study did not collect information on participants’ educational background or workplace setting (e.g., private or public clinic), which may also affect diabetes screening practices. Future research should consider these factors to provide a more comprehensive understanding. Further studies are required to increase the sample size and collect data in a more representative manner. Second, the cross-sectional study design prevented the examination of causal relationships with respect to attitudes, contributing factors, and diabetes screening practices and medical–dental collaboration. Prospective studies are required to confirm these findings.

Future research should include: (1) randomized controlled trials of educational interventions to improve screening practices, (2) longitudinal studies examining the impact of screening programs on diabetes detection rates, (3) cost-effectiveness analyses of dental-based screening programs, and (4) qualitative studies exploring organizational barriers to implementation.

## 5. Conclusions

This first nationwide study in Japan reveals significant gaps in diabetes screening practices among DHs, with implementation rates below 35% for most activities. Strong negative associations between confidence/knowledge deficits and screening practices identify clear intervention targets, while positive relationships with educational motivation suggest that targeted training programs would be effective. Current systemic barriers include asymmetric reimbursement structures favoring medical-to-dental referrals over dental-based screening and regulatory constraints on dental hygienist autonomy. Comprehensive reform involving curriculum enhancement, professional scope expansion, and financial incentive alignment is essential to realize the potential of dental settings as frontline diabetes screening venues. Given the bidirectional relationship between diabetes and periodontal disease, these improvements could significantly enhance early detection capabilities and strengthen medical–dental collaboration, ultimately contributing to better diabetes prevention and population health outcomes in Japan.

## Figures and Tables

**Table 1:** Background characteristics of participating DHs (N = 95).

Female	82	(86.3%)
Age (years) (*n* = 81)	44	±12.5
Clinical experience as a registered DH ^†^ (years) (*n* = 93)	20	±12.0
Time for diabetes patient education at dental clinics (min/patient) (*n* = 88)	22	±17.3
Employment status at dental clinics		
Full-time	74	(77.9%)
Part-time (single clinic)	18	(18.9%)
Part-time (multiple clinics)	3	(3.2%)
Experience of education in oral management of patients with diabetes	46	(48.4%)
Wish to participate in education regarding oral management of diabetes (*n* = 93)	69	(72.6%)

Values are presented as *n* (%) or mean ± standard deviation. Total N = 95; subtotals (*n*) vary by item due to missing data. ^†^ DH: dental hygienist.

**Table 2:** Exploratory factor analysis of DHs’ ^†^ attitudes towards diabetes screening and medical–dental collaboration in a dental setting.

	Factor 1	Factor 2	α
Factor 1: Perceived lack of confidence and knowledge in diabetes management			0.88
I am not confident in explaining the relationship between diabetes and periodontal disease to patients.	0.92		
I don’t know what to consider about diabetes.	0.85		
I usually practice oral management without thinking much about diabetes.	0.78		
I don’t have enough knowledge about diabetes.	0.74		
I don’t know dental hygienists’ role in medical–dental collaboration for diabetes.	0.73		
I don’t have enough time to explain the relationship between diabetes and periodontal disease to patients.	0.59		
I am confident in diabetes screening. *	0.55		
It is difficult for me to assess the level of glucose control from blood glucose levels and glycated hemoglobin.	0.44		
I can cope with hypoglycemia when patients experience hypoglycemia in a dental clinic. *	0.42		
Factor 2: Proactive approach to diabetes screening and interprofessional collaboration			0.77
I want to collaborate more with doctors and nurses to care for my patients with diabetes.		0.72	
I want to practice diabetes screening.		0.71	
Blood glucose monitoring is necessary in dentistry.		0.67	
I want to explain the relationship between diabetes and periodontal disease to my patients.		0.63	
It is necessary to assess diabetic status and periodontal treatment risks during oral management.		0.58	
I do not feel the need for medical–dental collaboration regarding diabetes treatment in daily practice. *		0.37	
Oral management education in medicine is necessary for medical–dental collaboration.		0.33	
Factor correlation	Factor 1	−0.28	

Values represent factor loadings obtained using the unweighted least squares method with promax rotation. Items with an asterisk (*) are reverse-coded. Factor 1 was labeled “Perceived lack of confidence and knowledge in diabetes management,” and Factor 2 was labeled “Proactive approach to diabetes screening and interprofessional collaboration.” Cronbach’s alpha (α) values are presented for the internal consistency reliability of each factor. Total N = 95. ^†^ DH = dental hygienist.

**Table 3:** Final items for assessing DHs’ ^†^ attitudes towards diabetes screening and medical–dental collaboration in a dental setting.

	Mean	±SD
Factor 1: Perceived lack of confidence and knowledge in diabetes management		
I am not confident in explaining the relationship between diabetes and periodontal disease to patients.	2.5	±0.9
I don’t know what to consider about diabetes.	2.4	±0.9
I usually practice oral management without thinking much about diabetes.	2.1	±0.8
I don’t have enough knowledge about diabetes.	2.7	±0.8
I don’t know dental hygienists’ role in medical–dental collaboration for diabetes.	2.3	±0.9
I don’t have enough time to explain the relationship between diabetes and periodontal disease to patients.	2.4	±0.9
I am confident in diabetes screening. *	1.9	±0.7
It is difficult for me to assess the level of glucose control from blood glucose levels and glycated hemoglobin.	2.5	±0.9
I can cope with hypoglycemia when patients experience hypoglycemia in a dental clinic. *	2.3	±0.9
Factor 2: Proactive approach to diabetes screening and interprofessional collaboration		
I want to collaborate more with doctors and nurses to care for my patients with diabetes.	3.0	±0.7
I want to practice diabetes screening.	2.9	±0.8
Blood glucose monitoring is necessary in dentistry.	2.7	±0.8
I want to explain the relationship between diabetes and periodontal disease to my patients.	3.4	±0.6
It is necessary to assess diabetic status and periodontal treatment risks during oral management.	3.6	±0.6
I do not feel the need for medical–dental collaboration regarding diabetes treatment in daily practice. *	1.7	±0.7
Oral management education in medicine is necessary for medical–dental collaboration.	3.3	±0.6

Attitudes were assessed through 15 items scored on a 4-point Likert scale: 4 = “Strongly agree,” 3 = “Somewhat agree,” 2 = “Somewhat disagree,” and 1 = “Strongly disagree.” Higher scores indicate stronger agreement with the statement. Values are presented as mean ± standard deviation. Items with an asterisk (*) are reverse-coded. Total N = 95. ^†^ DH = dental hygienist.

**Table 4:** Exploratory factor analysis of DHs’ ^†^ practice towards diabetes screening.

	Factor 1	Factor 2	α
Factor 1: Lifestyle background and physical sign assessment in diabetes			0.91
Polyuria	0.90		
Weight loss	0.85		
Fatigue	0.83		
Polydipsia	0.77		
Exercise habits	0.69		
Diet	0.68		
HDL-cholesterol and triglyceride levels	0.64		
Previous experience of hypoglycemia	0.57		
Thirst	0.49		
Body mass index	0.46		
Factor 2: Medical history and treatment status evaluation in diabetes			0.84
Ever been told your blood glucose was high		0.77	
The latest glycated hemoglobin		0.74	
Blood pressure		0.72	
Current diabetes treatment received		0.59	
Status of having a diabetes collaboration notebook		0.57	
Highest blood glucose level you have ever had		0.56	
Factor correlation	Factor 1	−0.48	

Values represent factor loadings obtained using the unweighted least squares method with promax rotation. Factor 1 was labeled “Lifestyle background and physical sign assessment in diabetes,” and Factor 2 was labeled “Medical history and treatment status evaluation in diabetes.” Cronbach’s alpha (α) values are presented for the internal consistency reliability of each factor. Factor correlation is also reported. Total N = 95. ^†^ DH = dental hygienist.

**Table 5:** Final items for assessing DHs’ ^†^ practice towards diabetes screening.

	Mean	±SD
Factor 1: Lifestyle background and physical sign assessment in diabetes		
Polyuria	1.8	±0.9
Weight loss	2.1	±0.9
Fatigue	2.1	±1.0
Polydipsia	2.1	±1.0
Exercise habits	1.9	±0.9
Diet	2.1	±0.9
HDL-cholesterol and triglyceride levels	1.7	±0.8
Previous experience of hypoglycemia	1.7	±0.8
Thirst	2.8	±1.0
Body mass index	1.6	±0.8
Factor 2: Medical history and treatment status evaluation in diabetes		
Ever been told your blood glucose was high	3.1	±0.9
The latest glycated hemoglobin	2.9	±1.1
Blood pressure	3.0	±0.9
Current diabetes treatment received	2.9	±0.9
Status of having a diabetes collaboration notebook	2.4	±1.0
Highest blood glucose level you have ever had	2.4	±1.1

Practices were assessed through 16 items scored on a 4-point Likert scale: 4 = “Always,” 3 = “Sometimes,” 2 = “Rarely,” and 1 = “Never.” Higher scores indicate more frequent implementation of the practice. Values are presented as mean ± standard deviation. Factor 1 was labeled “Lifestyle background and physical sign assessment in diabetes,” and Factor 2 was labeled “Medical history and treatment status evaluation in diabetes.” Total N = 95. ^†^ DH = dental hygienist.

**Table 6:** Multiple regression models of DHs’ ^†^ practices toward diabetes screening.

		Factor 1			Factor 2		
		β	95%CI		β	95%CI	
Attitudes							
Factor 1: Perceived lack of confidence and knowledge in diabetes management	9–36	**−0.38**	**−0.72**	**−0.23**	**−0.55**	**−0.63**	**−0.32**
Factor 2: Proactive approach to diabetes screening and interprofessional collaboration	7–28	−0.05	−0.55	0.34	0.11	−0.12	0.46
Gender	Female/Others	−0.15	−6.91	0.83	−0.03	−2.89	2.10
Clinical experience as a registered DH ^†^ (years)		**0.24**	**0.02**	**0.25**	0.02	−0.07	0.08
Employment status at dental clinics	Full-time/Part-time	−0.02	−3.64	3.04	0.07	−1.31	3.07
Time for diabetes patient education at dental clinics (minutes)		**0.26**	**0.03**	**0.18**	−0.09	−0.08	0.02
Desire to participate in education regarding oral management of diabetes	Yes/no	0.11	−1.47	4.90	**0.21**	**0.24**	**4.38**

Attitudes were assessed through two factors: Factor 1 “Perceived lack of confidence and knowledge in diabetes management” (9–36 points) and Factor 2 “Proactive approach to diabetes screening and interprofessional collaboration” (7–28 points). Practices were assessed through two factors: Factor 1 “Lifestyle background and physical sign assessment in diabetes” and Factor 2 “Medical history and treatment status evaluation in diabetes.” Multiple regression analysis was conducted with attitudes and practices factors as dependent variables. β represents standardized partial regression coefficients with 95% confidence intervals (CI). Statistical significance levels: *p* < 0.05. Bold values indicate statistically significant results. Gender was categorized as Female/Others. Employment status was categorized as Full-time/Part-time. Desire to participate in education was coded as Yes/No. Total N = 95. ^†^ DH = dental hygienist.

## Data Availability

The data presented in this study are available upon request from the corresponding author. The data are not publicly available due to privacy or ethical restrictions.

## Abbreviations

The following abbreviations are used in this manuscript:

DH	Dental Hygienist
